# Policymaking ‘under the radar’: a case study of pesticide regulation to prevent intentional poisoning in Sri Lanka

**DOI:** 10.1093/heapol/czt096

**Published:** 2013-12-20

**Authors:** Melissa Pearson, Anthony B Zwi, Nicholas A Buckley, Gamini Manuweera, Ravindra Fernando, Andrew H Dawson, Duncan McDuie-Ra

**Affiliations:** ^1^South Asian Clinical Toxicology Research Collaboration, Faculty of Medicine, University of Peradeniya, Sri Lanka and School of Social Sciences, Faculty of Arts and Social Sciences, University of New South Wales, Randwick NSW, 2502 Australia, ^2^Health, Rights and Development (HEARD@UNSW), School of Social Sciences, Faculty of Arts and Social Sciences, University of New South Wales, Sydney, Australia, ^3^South Asian Clinical Toxicology Research Collaboration, Prince of Wales Hospital Clinical School, University of New South Wales, NSW, Australia, ^4^Scientific Support Branch, Secretariat of the Basel, Rotterdam and Stockholm Conventions, United Nations Environment Programme, ^5^South Asian Clinical Toxicology Research Collaboration, University of Peradeniya Sri Lanka and Department of Forensic Medicine, Faculty of Medicine, University of Colombo, Colombo, Sri Lanka, ^6^South Asian Clinical Toxicology Research Collaboration, University of Peradeniya Sri Lanka and NSW Poisons Information Service, Westmead Childrens Hospital, Sydney, Australia and ^7^School of Social Sciences and International Studies, Faculty of Arts and Social Sciences, University of New South Wales, Sydney, Australia

**Keywords:** Suicide, pesticides, policy analysis, evidence-based policy, health policy, agriculture, prevention, developing countries, Sri Lanka

## Abstract

**Background** Suicide in Sri Lanka is a major public health problem and in 1995 the country had one of the highest rates of suicide worldwide. Since then reductions in overall suicide rates have been largely attributed to efforts to regulate a range of pesticides. The evolution, context, events and implementation of the key policy decisions around regulation are examined.

**Methods** This study was undertaken as part of a broader analysis of policy in two parts—an explanatory case study and stakeholder analysis. This article describes the explanatory case study that included an historical narrative and in-depth interviews.

**Results** A timeline and chronology of policy actions and influence were derived from interview and document data. Fourteen key informants were interviewed and four distinct policy phases were identified. The early stages of pesticide regulation were dominated by political and economic considerations and strongly influenced by external factors. The second phase was marked by a period of local institution building, the engagement of local stakeholders, and expanded links between health and agriculture. During the third phase the problem of self-poisoning dominated the policy agenda and closer links between stakeholders, evidence and policymaking developed. The fourth and most recent phase was characterized by strong local capacity for policymaking, informed by evidence, developed in collaboration with a powerful network of stakeholders, including international researchers.

**Conclusions** The policy response to extremely high rates of suicide from intentional poisoning with pesticides shows a unique and successful example of policymaking to prevent suicide. It also highlights policy action taking place ‘under the radar’, thus avoiding policy inertia often associated with reforms in lower and middle income countries.

**KEY MESSAGES**

- The regulation of pesticides in Sri Lanka over a period of 20 years reduced the mortality from suicide and offers an illustration of intersectoral collaboration to prevent avoidable deaths.
- The strong local ownership of the problem was established as local researchers and clinicians documented the social and health care burden and this led to a window of opportunity for policymaking.
- A strong network allowed a dominant frame of the problem to emerge and this facilitated action to be taken.
- The technical nature of decision making and networks between research communities in health and agriculture allowed policy action to continue free from political interference, ‘under the radar’.

## Introduction

This article examines the evolution of policy decisions on suicide prevention within the context of the regulation of pesticides in Sri Lanka. Suicide in Sri Lanka is a major public health problem, and in 1995, the country had one of the highest rates of suicide worldwide—47 per 100 000 population (Ratnayeke 1996). Recent analysis of the incidence of suicide has shown a substantial decline from the peak in 1995 (male 80 and female 28) to 24 per 100 000 in 2005 (male 37 and female 10) (Gunnell *et al.* 2007).

The incidence increased dramatically in the late 1970s from 17.43 per 100 000 in 1977 to 46.94 per 100 000 in 1995 followed by three steep reductions. Over a 30-year period, regulation of pesticides has been shown to be more strongly linked to declining incidence rates than employment, divorce, overall pesticide use and civil conflict (Gunnell *et al.* 2007). Although many authors have noted other factors that may have contributed to the decline such as improvements in transport, changes to less lethal methods and medical management (Roberts *et al.* 2003; Eddleston *et al.* 2005; Silva *et al.* 2012), it is widely accepted that the regulation of pesticides contributed to reduced mortality from suicide (Gunnell *et al.* 2007; Ministry of Health and WHO 2007; Chen *et al.* 2012; De Silva *et al.* 2013).

This success in reducing the burden of suicide is both remarkable and unique in Asia. Despite a similarly high burden related to self-poisoning with pesticides in other Asian countries notably India and China (Phillips *et al.* 2002; Patel *et al.* 2012), the effectiveness of policy to regulate pesticides is less apparent. Analysis of the successes of this Sri Lankan policymaking process could be of value to other countries.

### Suicide prevention

Policy responses to complex and multi-faceted social problems, such as suicide, require intersectoral collaboration, given the variety of social, cultural and political determinants. The majority of literature on suicide prevention is focused on high income countries (HIC). In the Asian region, efforts around suicide prevention have focused on the importance of pesticides (Beautrais 2006; Hendin *et al.* 2008; WHO 2009; Yip *et al.* 2012). Recommendations for suicide prevention in Asia have often included increasing community-based responses, restricting access to lethal means, reducing harmful use of alcohol, prevention and treatment of depression and improving how the media portrays this problem (World Health Organization 2008; Chen *et al.* 2012). Restricting access to lethal means and more specifically the regulation of pesticides has been noted as an important strategy; this has been reinforced by evidence from Sri Lanka (Roberts *et al.* 2003; Gunnell *et al.* 2007).

### Context of suicide prevention in Sri Lanka

Suicide in Sri Lanka has been described as manifesting differently from HIC in both method and intention (Pearson *et al.* 2013). The majority of deaths are attributed to intentional ingestion of pesticides, commonly found within households in rural communities (Gunnell and Eddleston 2003; Eddleston *et al.* 2005; Konradsen *et al.* 2006). Suicide and self-poisoning continue to be one of the main causes of admission to hospital and one of the leading causes of death (Eddleston *et al.* 2005). In Sri Lanka responses have included establishing a Presidential Committee (see Box 1), legislative changes and improved clinical management.

**Box 1 The Presidential Committee (formed in 1997) developed a National Suicide Prevention Strategy in December 1997 which sought to**

- reduce easy access to lethal methods;
- promote research on reducing the lethality of pesticides in use;
- educate the public on less harmful use of pesticides;
- create a culture which discourages suicides;
- ensure survival after poisoning; and
- remove legal barriers to the correct handling of those at risk (Government of Sri Lanka 1997).

The focus of the national strategy and action plan highlighted the importance of controlling pesticides for suicide prevention and the necessity for intersectoral collaboration between agriculture and health.

### Structure of pesticide regulation

Agriculture maintains a prominent place within society despite its declining share of gross domestic product from 28% in the early 1980s to 20% in 2000. As a sector, 45% of households nationally remain engaged in agriculture (World Bank 2003). Pesticides are commonly used in agriculture and became widespread in Sri Lanka during the 1980s (Fernando 1993; FAO 2005). Pesticides are used for crops (rice, fruit and vegetable) and in the plantation sector. All pesticides are imported, costing Sri Lanka around 1350 million rupees ($12.3 m US Dollars) in 2008 (Centre for Environmental Justice 2006).

The regulation of pesticides is mandated through the Control of Pesticide Act 1980. The Act provides for regulation of the import, formulation, use, sales, packaging, labelling, storage and transport of pesticides. In addition, the Act established structures for its implementation. Within the Department of Agriculture (DoA), the Office of the Registrar of Pesticides (ORP) has responsibility for ensuring administration of all the registration procedures. The position of Registrar of Pesticides (RoP) must be occupied by a professional with postdoctoral qualifications in agricultural sciences.

The Pesticides Technical Advisory Committee (PeTAC) established in the Act is mandated to provide technical advice and decisions regarding the registration and regulation of pesticides. The PeTAC comprises 15 members including 10 permanent members: Director General of Agriculture (Chairperson), RoP, Director General of Health Services, Commissioner of Labour (Occupational Health), Director General Central Environment Authority, Government Analyst, Directors of Agricultural Research Institutes (Tea, Rubber and Coconut) and the Director General of Sri Lanka Standards Institute. In addition, there are five advisory positions nominated by the Minister of Agriculture, each serving for 3 years. In 2008, these were held by an ex-RoP, a chemistry professor from the Agriculture Faculty at University of Peradeniya, a weed scientist from the department, an official from the Department of Customs and one from the Attorney General’s Department.

Since the inception of the Control of Pesticides Act 1980, the DoA has embarked on a concerted programme to regulate the most toxic pesticides (Table 1). These regulations in sales, formulation, import restrictions and marketing have been associated with reductions in overall mortality from intentional self-poisoning (Gunnell *et al.* 2007; Roberts *et al.* 2003). In 2008, the DoA announced a phased withdrawal of three more pesticides (paraquat, dimethoate and fenthion) based on strong evidence of the high case fatality associated with their misuse in rural communities (Dawson *et al.* 2010).

**Table 1:** Import bans of pesticides in Sri Lanka 1970–2008

Year	Chemical	Reason for ban
1970	Endrin, toxaphene, chlordimeform, thallium	Import policies
1976	DDT	Environmental concerns
1984	Parathion, 2-4-5T, arsenic, captafol, lepatophos, HCH lindane, mercury compounds	Environmental concerns
1986	Aldrin, heptachlor	Environmental concerns
1992	Dieldrin	Environmental concerns
1994	Atrazine	Groundwater and subsoil contaminant
1995	Monocrotophos, methamidaphos, dichloropropane, aldicarb, pentachlorophenol, quintozene	Removal of all Class Ia and b
1996	All POPs—chlordane	Environmental concerns
1997	Endosulfan	Environmental contamination of groundwater and suicide
2008	Paraquat, dimethoate, fenthion^a^	Suicide

^a^Phased bans over 3 years.

The regulation of pesticides in Sri Lanka is seen as exemplary in the region with the link to policymaking on intentional self-poisoning being seen as unique in many ways (Wiebers 1993). This case study examines how this policy response unfolded. Our study addressed three main questions: how did the issue come to be identified as a problem? What explains this apparent success? How did the social policy response, with its intersectoral character, come about?

## Methods

**Figure 1:**
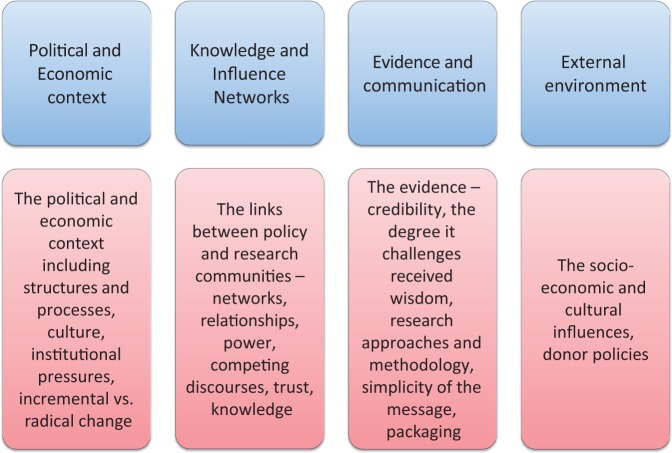
Modified Research And Policy In Development (RAPID) research-to-policy framework.

An explanatory case study methodology was used and employed two specific tools: a timeline and in-depth interviews. A narrative historical description was developed using published material to explore timelines with informants across a range of factors including macro-political context (policies, international conventions and national political events), national context of policy formation (agenda, evidence and appointments), implementation and policymakers (actors and policymakers) (Court and Cotterrell 2004). A semi-structured interview framework was developed based on the context, evidence and links model developed by the Overseas Development Institute (Crewe and Young 2002) to investigate the impact of research on policy (Crewe and Young 2002). The original framework was slightly modified in two ways: first by renaming ‘context’ as ‘political and economic context’ and second the ‘links’ category was renamed ‘knowledge and influence networks’. These changes were made to facilitate a clearer understanding of concepts presented in our research (Figure 1). The interviews also included a tool to collect data about links and relationships to formally examine the importance and influence of stakeholders.

Participants were selected through a snowball procedure (a systematic non-probabilistic sample) to ensure wide recruitment (Mays and Pope 1995). An initial list of informants was developed through review of documents. This list was then reviewed by a small research group who nominated additional informants. Each participant was also given the opportunity to nominate further informants.

Interviews were taped and transcribed. Notes from documents, transcripts and stakeholder discussions were coded in NVIVO 8 (Mays and Pope 1995; NVivo 2009). Data were analysed based on a framework approach (Ritchie and Spencer 2002) of familiarization, identifying a thematic framework, indexing, charting and mapping/interpretation. All participants were fluent English speakers, the language in which interviews were conducted.

Purposive selection, use of grounded theory, triangulation, reflexivity and respondent validation were included in the design of this study in keeping with good practice for qualitative studies (Pope *et al.* 2000; Gilson *et al.* 2011) and to address bias, internal validity, and confirm interpretations (Table 2). Informed consent was obtained in writing from all participants including consent to be audiotaped.

**Table 2:** Application of quality procedures to pesticide policymaking study

Quality procedures	Concerns addressed	How procedures applied
Purposive selection	Bias	Use of local research team and snowball procedure to check ‘outliers’
Grounded theory	Original theorizing	Development of emergent themes distinct from interview framework
Triangulation	Confirmation or refutation of internal validity	Use of interviews, review of documents, papers and stakeholder tables
Reflexivity	Validity of interpretations	Disclosure of researchers’ position for readers
Respondent validation	Confirmation or refutation of interpretations	Discussion of outcomes, iterative approach to interview framework, review of paper

^a^Adapted from Mays 2000 and Barbour 2001 (Mays and Pope 1995; Barbour 2001).

Selected quotes were checked with participants for consent to publish. In the text that follows, participants have been identified by their general roles to facilitate understanding of verbatim quotes except where this could identify the participant. The study received ethical clearance from the University of Ruhunu Sri Lanka (July 2008) and University of New South Wales, Australia (September 2008 HREC 08265).

The research team comprised a postgraduate research student, a programme director of the South Asian Clinical Toxicology Research Collaboration (SACTRC), the RoP and a professor of Forensic Medicine from Colombo University. This team helped design the study, review the outcomes and write the paper. In addition, supervision of the study was undertaken by three academics at University of New South Wales who assisted in the design of the study, analysis of the results and writing.

The social location of researchers and their personal qualities, values, gender, ethnicity and class identities can have an important influence on the results and analysis of research findings (Richards and Emslie 2000; Hewitt 2007). The use of the local researchers to review findings and outside researchers to refocus methods, and interpret results allowed for alternative views to be corroborated and ensure appropriate conclusions were drawn.

## Results

Fourteen key informants were interviewed including two Pesticide Registrars (current and former), agricultural researchers, clinicians, health researchers, a local non-governmental organization representative, industry representatives and a representative from an international agency. The characteristics of participants ranged according to gender, sector, role and nationality (Table 3).

**Table 3:** Characteristics of participants in pesticide policymaking study

Category	Characteristic	No.
Gender	Male	11
	Female	3
Sector	Agriculture	5
	Health	8
	Voluntary	1
Role	Civil servant	2
	Academic	5
	Clinician/academic	3
	Non- Government Organisation	2
	Industry	2
Nationality	Sri Lankan	9
	International	2

Here, we present a brief description of the identifiable phases along with key background information.

### History of pesticide policymaking

**Figure 2:**
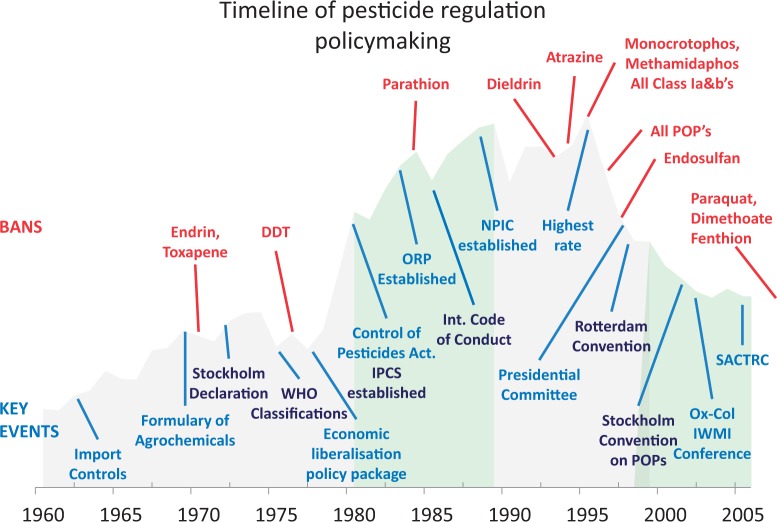
Timeline of events related to pesticide regulation in Sri Lanka (1960–2008).

A number of policy decisions were made to restrict the import and sales of specific agents during the period under study (Table 1). The first regulatory actions were two import restrictions on pesticides prior to the enactment of the Control of Pesticides Act No. 33 of 1980. Following the establishment of the ORP, an additional six restrictions were made for a number of chemicals. We identified four discernible phases of policymaking in relation to pesticide regulation from 1960 to 2005. These coincide roughly with the decades seen in Figure 2 and are described in summary (Table 4) and detail with their key features below.

**Table 4:** Characteristics of the phases of policymaking related to pesticide regulation in Sri Lanka 1960–2008

	Political and economic context	Knowledge and influence networks	External environment	Evidence and communication
Pre-1980s	Import controls to protect foreign exchange 1976 economic liberalization package—proliferation of agro-chemicals	Government of Sri Lanka (GoSL) asks for assistance from FAO to draft Control of Pesticide Act DoA participating in international chemical safety agreements discussions	Rising concern about chemical safety Green revolution–synthetic	Local agronomy research into appropriate pesticides to rationalize use Links to international research
1980s	Tabling and ratification of Control of Pesticides Act ORP established—strong technical facility free from external pressure Process of registering chemicals	NPIC established with assistance of WHO and International Development Research Centre (IDRC) Links between NPIC and ORP begin Strong links between ORP and International Program on Chemical Safety (IPCS)	International Code of Conduct for the Distribution and Control of Pesticides Establishment of multi-agency IPCS	Evidence of problem with self-poisoning starts to emerge in hospital settings Occupational health paper outlines the problem of suicide
1990s	Sri Lanka cited by WHO as having one of highest rates of suicide in the world Presidential Committee of prevention of suicide set up Bans on some pesticides, predominantly resulting from chemical safety agenda	Local links between health and agriculture strengthened Informal and formal mechanisms develop	WHO quarterly statistics published and highlights high rate of suicide in Sri Lanka Interaction continues on IPCS-setting chemical safety agenda	Local evidence of problem strong Crossover from clinical medicine to risk factors, geographic, methods
2000s	Delegated policymaking Technical responses supported	Epistemic community becomes powerful and pervasive Links with industry strengthen support for decision making	Little direct involvement; most external presence manifest through international researchers and their linkages	International research collaborations strengthen capacity for research Engagement between policymakers and researchers strong Industry collaborate with research

### Pre-1980: reactive policy

The early period of pesticide regulation was dominated by political and economic considerations. In 1964, a range of restrictions were placed on the import of pesticides to conserve foreign currency. The DoA in 1970 developed the Formulary of Agrochemicals with a view to recommending the most effective crop pesticides and limiting the number available. In 1977, an economic liberalization policy package was implemented and limited state intervention in the market. A number of participants highlighted the increasing availability of pesticides following these reforms.

In the 1970s, there was heightened international awareness concerning environmental and human health hazards associated with pesticides. During this period, the DoA sought assistance from the Food and Agriculture Organization (FAO) to draft legislation relating to pesticide controls. In 1980, the Government of Sri Lanka tabled the Control of Pesticides Act No. 33 of 1980. The Act mirrors the International Code of Conduct for the Distribution and Use of Pesticides that was not ratified by the FAO governing body until 1985. The collaboration between the DoA and the FAO during a period in which both were drafting policy ensured that technical structures were embedded in the legislation. An agriculture official noted the importance of parity between the international and the national frameworks.

“*There is significance in this because even if we now look at our Law, it is very, very similar. There are lots of similarities between our Law and the FAO Code of Conduct because the original Technical Assistant posts were through the FAO’s.”* Agriculture official

### 1980–89: institution building and capacity development

The second phase was marked by a period of local institution building and capacity development. Following the ratification of the Control of Pesticides Act in 1980, the ORP was established in 1983 and implementation of the Act commenced. The initial task of the Office was to register all agrochemicals in use in Sri Lanka at the time. The appointment of the RoP was closely linked to the University of Peradeniya Faculty of Agriculture and this established a technical basis for policymaking. The three appointments to the post of RoP have been widely respected for their scientific qualifications as articulated below.

“*Dr Nalini de Alwis (Ex-Registrar of Pesticides) was the former Deputy Director (Research) at Gannoruwa Research Station; … according to the administrative hierarchy at this time; she was positioned next to the Director. So this post was a very high status position on the research side. She was a very highly recognized entomologist, who had completed her PhD in [the] US and was highly respected as a research Scientist.*” Agriculture Official

“*A brilliant scientist.”* Industry Representative’s comment on former Registrar of Pesticides

The emerging recognition of the problem of suicide and self-harm with pesticides had its origins in the health services. A community study in 1982 (Jeyaratnam *et al.* 1982) highlighted pesticide related problems in rural areas.

“*The study was a bit of an eye-opener; probably, one of the earliest use of epidemiological research and also at national level*.” Local Academic

The evidence in the 1980s was primarily generated from medical units as they struggled to effectively treat the large numbers of people being admitted as a result of poisoning and self-harm. Journal articles (Jeyaratnam *et al.* 1982; Senanayake and Johnson 1982; Ganesvaran *et al.* 1984; Ariyananda 1986; Senanayake 1986; Senanayake and Karalliedde 1986, 1988; Fernando 1988; Hettiarachchi *et al.* 1988; Karalliedde and Senanayake 1988; Hettiarachchi and Kodithuwakku 1989), conferences and medical society meetings were used to highlight the problem. A group of physicians at the University of Peradeniya were central and their early links with the Faculty of Agriculture established relationships that facilitated future policy collaboration.

“*On reflection I think the work at Peradeniya at least played a fairly significant role of highlighting not only the incidence but also the problems and the clinical profiles of pesticide poisoning in the Sri Lankan medical community.*” Local Academic

The collegial networks of these clinicians and academics helped forge academic linkages between health and agriculture.

The National Poison Information Centre (NPIC) was opened in 1988 with funding from the International Development Research Centre in Canada. The Ministry of Health appointed a respected professor from Colombo University to the post of director and this established a medical focal point for poisoning and a platform for advocacy. The formal and informal links between the NPIC and ORP facilitated future intersectoral collaboration.

### 1990–99: emerging recognition of the problem

During the third phase, the problem of self-poisoning with pesticides dominated the policy agenda and links between stakeholders, evidence and policymaking at local level gained legitimacy. The problem of suicide and its relationship to the easy availability of pesticides was different from the patterns of suicide observed elsewhere, as highlighted below:

“*Since these pesticides are commonly available in all families, the first thing that they reach for in a quarrel is a bottle of pesticide.*” Community Worker

The tipping point from the accumulated evidence was the publication of World Health Organization (WHO) statistics on suicide in 1995 which led to recognition that Sri Lanka had one of the highest rates of suicide in the world (Ratnayeke 1996). This fact caused embarrassment to the Government, highlighted by several participants:

“*I think the most important issue at that time was the political commitment. It was seen as an embarrassment. It had been publicly profiled; it was clearly an embarrassment to the healthcare service but even beyond that it became regionally known that Sri Lanka was a hotbed for suicide and that pesticides were the leading cause.*” Local Academic

“*Everywhere it was reported that Sri Lanka had the highest suicide rate in the world due to pesticides and so that really rang alarm bells and you should do something about it.*” Agriculture Official

Following awareness of high rates of suicide in Sri Lanka, a Presidential Committee was appointed (1997). The Committee met monthly for a year and produced a National Policy and Action Plan on Prevention of Suicide released in 1998 (Government of Sri Lanka 1997). The influence of this high-level commitment was profound.

“*I think it was the President that made a big difference.*” Local Academic

This also signalled an important shift within the DoA in relation to how it viewed suicide; it had previously been viewed as a social problem beyond their remit. However, links between health and agriculture connected the problem to the easy availability of pesticides and specifically to their sales, marketing and promotion.

“*I think some of the Managers (Agriculture) and industry realised all of a sudden that you couldn’t separate the two (pesticides and suicide). That so far, it was not just a reflection of an impulsive problem as a social issue but there were so many aspects of what products were available, how they are marketed, how it is packed, how it is sold, how it is promoted and how it is stored that influence whether or not a vulnerable person can and will use it.*” International Academic

However, following the publication of the national strategy, many informants noted the limited subsequent political engagement. Although high profile involvement was not sustained, engagement at this level legitimized actors and networks to continue policy activity and high-level support was neither sought nor required.

### 2000–08: evidence-informed policymaking

The fourth phase was characterized by strong local capacity for policymaking informed by evidence developed in collaboration through a powerful network of stakeholders. This epistemic community, described elsewhere (Pearson *et al.* 2010), generated evidence through surveillance and research, and sustained the links and relationships consolidated through ongoing communication involving individuals and institutions, both within and outside, Sri Lanka.

“*Our role has been to support the RoP with technical information to support decision making*” International Academic

“*They (plant protection scientists and medical academics) developed a collaboration and they were a very strong group that of course had an impact on recent developments … they are very professional people and that really made a difference.*” International Academic

The significant local engagement among researchers and regulators enabled further collaboration.

“*It was the fact that previous activity and policy suggested in Sri Lanka [that] they were not just interested in pesticides but also that they were concerned about suicide. The way that the problem and policy intersected is really one of the reasons we actually came here. We were looking to do something about this problem regionally, and we figured that we should come to a country, which looked most likely to succeed.*” International Academic

In 2008, PeTAC made the decision to withdraw ‘paraquat’, *‘*fenthion’ and ‘dimethoate’ on the basis of evidence of harm caused by the intentional poisoning with pesticides. This decision was linked to the evidence generated in the research community.

Despite the success in Sri Lanka, the epistemic community found it difficult to influence international agendas and a number of participants expressed frustration at trying to engage suicide prevention and chemical safety communities within other agencies, notably the WHO:

“*We had some engagement with the WHO at the time on water management, disease pest control, malaria, and Japanese Encephalitis. Never in any of the communication, no matter how hard you tried … In Geneva or New Delhi, they could not be bothered at all.*” International Academic

In addition, there was another important network that evolved around pesticide control—primarily the ORP and industry representatives. Most of this group had studied at the Agriculture Faculty at Peradeniya and it was a collegiate environment with a common sense of purpose and responsibility to act.

“*The pesticide industry is working responsibly with the medical profession as well as the pesticide regulators.*” Local Academic

“*Suicide is a problem; we see that; the reports came from the National Poison Information Centre through Professor Ravindra Fernando. We see that pesticides are an easy way to attempt suicide; so we also have a responsibility.*” Industry Representative

These unique relationships were cited by a number of participants as unique and beneficial to the control of pesticides. However, several participants noted the lack of visibility and voice from rural constituencies. Several participants felt that they had an obligation to ensure the views of farmers were considered, echoing the responsibility felt towards rural communities.

“*Yes, the farmers are a very passive stakeholder in Sri Lanka … So, they are not really represented or significant in this whole thing. So, I feel sorry about this and I always try to represent them in my dealings.*” Government Official

However, one participant noted that the behaviour of farmers (drinking pesticides) has had a powerful influence on policy:

“*Well the farmer behaviour patterns have influenced policy on suicide. They have misused pesticides, as they are meant for crops, not ingestion. So farmers’ behaviour—the deaths—are what made everybody sit up and look.*” Community Worker

## Discussion

The Sri Lankan policy response to extremely high rates of suicide from intentional poisoning with pesticides demonstrates successful policy action. A major problem was identified and effective policy action followed. We explore what, how and why this occurred.

### How can we explain the apparent success?

We identified four phases in the policy response to pesticide regulation as an intervention to address suicide in Sri Lanka. The first phase (pre-1980) resembles many policy environments where national and international concerns coincide to generate policy activity. The second highlights institution building in providing solid foundations for future policymaking activities. A high-profile key event—the Presidential Commission—dominates the third phase. This event would provide future legitimacy for policymaking. Finally, the fourth phase highlights the contributions of evidence to inform policymaking as well as demonstrating mechanisms to facilitate intersectoral collaboration.

Local political concerns and external influences were the basis for decision making in the first phase (pre-1980). This resembles a ‘muddling through’ approach to policymaking (Lindblom 1959) in which decisions were directed to specific strategies. The foreign exchange shortage was an example of ‘policy change under crisis’ (Walt 1994), with economic considerations driving policy change even in a quite specific area such as pesticide imports. The proliferation of pesticides following economic liberalization compelled the DoA to establish systems for determining which chemicals should be locally available. The institutional arrangements of the ORP as an integral part of the DoA assist its establishment and secure its long-term funding. International agencies are able to influence national concerns about pesticides during this period and the FAO was approached to provide expertise in drafting legislation. Policy activity was largely reactive, but signalled the need for the development of systems to respond.

The second phase (1980s), in retrospect, can be shown to have focused on capacity building, developing personnel, structures and mechanisms (e.g. legislation) to create a robust system. External funding is used to support the development of key institutions. Even during this phase there were some unique features: the DoA and the Government of Sri Lanka took a leading role in developing legislation to control pesticides even prior to the publication of the International Code of Conduct for the Distribution and Use of Pesticides. Most other countries developed legislation only after publication of the Code (FAO 2005). The desire to be a role model and to incorporate the best available evidence helped provide a strong foundation for future policymaking.

During the third phase, the problem of suicide and self-poisoning forced its way onto the agenda. Kingdon postulates that issues get onto the agenda when the problem, politics and policy streams intersect to provide a ‘window of opportunity’ (Kingdon 1984). This ‘window of opportunity’ resulted from the publication of the WHO global suicide rates. Policy proposals were actively debated as the problem of suicide embarrassed Sri Lanka. Thus policy did not proceed in an orderly fashion from identification of the problem to the consideration of alternatives (Walt 1994). Rather the problem, that was identified as early as 1982, was followed by a protracted period of developing momentum until a window of opportunity presented.

The period following the establishment of the Presidential Committee, however, is less well explained by current theories on policymaking. While on a superficial level, it is possible to see the establishment of the Committee and the publication of a national suicide prevention strategy as outcomes from the window of opportunity. Previous policy analysis (Jones *et al.* 2009) has stressed the importance of political commitment and in our study this long term commitment was not observed. We believe, however, that the short period of political commitment was sufficient to establish and legitimize the personnel and institutions that could take forward future policy decisions.

High-level political involvement is also absent from policymaking during the final phase. It appears that the PeTAC functions independently and decisions, free from political interference, were considered within a technical domain with evidence both being commissioned and considered informing action. An epistemic community formed between agricultural scientists, public health practitioners, and clinical toxicologists, the latter both from within and outside Sri Lanka. This community was, and in 2010 continued to be, well defined within this policy domain, with a tightly integrated group, limited in number, with high continuity and members having roughly equal status and power (Pearson *et al.* 2010). Researchers both national and international were very active and their interaction with policymakers defines the period. PeTAC was also free from the commercial pressures of agrochemical manufacturers although the ORP maintains a constant dialogue with industry and fosters a joint sense of purpose. Thus, policy activity can be characterized as informed by evidence (Bowen and Zwi 2005) rather than being an expression of an authoritative choice (Colebatch 2002) form of decision making.

Policymaking over this period can be seen to pass through a range of phases: muddling through, capacity development, agenda setting and evidence-informed. The success of the policy decisions in the latter period can be traced through the historical development of policy through these phases.

### How did it come to be identified as a problem?

To understand how the issue of intentional self-poisoning came to be identified as a problem for agricultural policymakers, it is useful to consider social construction of policy (Colebatch 2002) through the framing of policy dialogues (Rein and Schön 1996). Framing employs ‘storylines that set a specific train of thought in motion, communicating why an issue might be a problem, who or what might be responsible for it, and what should be done’ (Nisbet 2009). Issues are contested in this perspective, and some ideas gather traction as policy problems whereas others are left unaddressed (Hajer 1995; Roggeband and Verloo 2007). Early in the period under study, the problem of self-poisoning with pesticides is perceived to be a social problem and not an issue for agriculture policymakers. As the burden on medical services and associated high case fatalities became apparent, interactions between clinicians and the Office of the Pesticide Registrar began to address the problem. The link between pesticides and suicide was already well established by the time the Presidential Committee formed.

The National Strategy (1997) reflects the dominance of this framing of the problem that clearly linked the problem of suicide with pesticides. Pesticides are directly related to four of the six action points. The other two related to improved medical management following poisoning and creating a culture that discourages suicide. The link between suicide and mental health was not seen to be as important in this context, and this narrative is less apparent. The contested nature of the academic literature on the role of mental health may have contributed to this (Pearson *et al.* 2013).

Although these theories and frameworks help to explain aspects of the unique policy change explored here, this policy story can also be construed as ‘problems looking for solutions’. The solutions proposed and considered are those around which there is limited contestation, and solutions can be implemented—the tools and approaches are available. Some of the difficult dimensions are largely ignored, especially the underlying causes of suicide, as the avenues for responding to them are far less obvious.

Although this helps action to be taken particularly in regulation, it also allowed more complex narratives to be ignored, and alternative solutions, more contentious and contested, to be avoided. The inability to generate support and the associated policy traction from a wider range of participants can be seen, at least in part, to reflect the difficulty of developing convincing narratives for addressing complex social problems (Lu 2006). Thus, future policy change may be more fragile as easier issues are addressed, and now the more complex dynamics need to be considered.

### How did the social policy response come about?

This period of policymaking appears to have operated on middle ground; relatively free from pressure from above (national or international) as well as from below. Exploring the characteristics that allowed this pattern to emerge benefits from the framework of policy change suggested by Grindle and Thomas (1989). They identified four factors as being influential in driving policy and institutional choice in developing countries: technical analysis, bureaucratic motivation, political stability and support, and international leverage.

Technical analysis has clearly been a strong and persuasive influence on policy activity and change in this period. In addition, there is widespread support culturally for technical and policy solutions to social problems. Bureaucratic motivation (and politics) in developing countries has been often shown to influence decision making and policy choices especially if this has implications for power and position of individuals, units or departments (Grindle and Thomas 1989). The influence of the position and ORP helps to explain some aspects of this. The Control of Pesticides Act 1980 provided a powerful mandate to the PeTAC and ORP and delegated authority for decision making to a technical committee, strictly controlled in terms of its composition, credentials and scope of influence. There was no need to fight for status and power as the technical basis for decision making was, and continues to be, enshrined in the Act. Thus, bureaucratic politics and bureaucrats themselves were supportive rather than being a barrier to change. They operated to facilitate change and reform and can be credited with a degree of ‘benevolence’ that created space from politics and legitimacy in policymaking.

Political stability was not at issue in this example. Politics in many countries is often considered as an obstacle to evidence-informed policymaking; its relative absence in this study may have contributed to the success. The lack of a local pesticide manufacturing sector removed one potential pressure as there was no obvious economic rationale for lobbying political actors. The lack of engagement by farmers is, however, simultaneously startling and yet in keeping with some form of ‘benevolent’ policymaking in which a bureaucratic and political commitment to promoting community and social benefits is, at some level, present. An earlier publication highlighted that the minimal community reaction or pressure resulting from the changes to the availability of pesticides, suggesting a passive acceptance of policymaking (Pearson *et al.* 2009). In addition, the ongoing civil conflict consumed political energies and agendas, allowing other issues to be taken forward with limited high-level political engagement or interference.

International leverage has been described as the persuasiveness of international pressure for reform (Grindle and Thomas 1989). In this case study some of the early policy decisions to create structure and legislation can be seen to be tied, although not directed by international influences. The main influences were environmental and safety concerns related to pesticides. In the policy problem associated with high numbers of suicides, international agencies are largely absent. Thus, local ownership of the problem and responses proceed without becoming politicized. In this study, political stability was maintained and considerable progress made by keeping policy activity ‘under the radar’.

## Limitations

This study has highlighted the specificity of the policy response to pesticides and suicide in Sri Lanka. However, the study may be limited by the availability of historical figures for interview and memory recall associated with historical analysis. There has been a clear motive for respondents to recognize the importance of suicide as being on the agenda for policy formulation. Recall bias may have conferred earlier recognition of the problem than perhaps was evident at the time but in our study there was also a clear change noted in the narrative of policymaking.

The limited range of participants especially women reflects the status of women in higher levels of office. The limited number of informants was nevertheless appropriate to such a specific policy event and the use of iterative processes to conceptualize and reconceptualize the policy story was in keeping with good practice for rigorous qualitative investigations (Mays and Pope 1995; Gilson *et al.* 2011).

A further limitation as a result of the researchers’ position may have restricted the identification of other important and less well articulated socio-cultural values. Respondent validation and use of local collaborators to reflect on findings attempted to address these issues. In addition, research supervision from outside the involved groups help to reflect on the findings and contribute alternative explanations. However, research by international researchers in lower- and middle-income countries must also be considered to have had some impact on the interpretation of the results.

## Conclusion

Sri Lanka offers an interesting example of effective policymaking on self-poisoning with pesticides from which wider policy lessons can be drawn. The regulation of pesticides in Sri Lanka over a period of 20 years has reduced the mortality from suicide; policymakers in agriculture responded to a perceived crisis. The problem of suicide in Sri Lanka grew in importance as local researchers and clinicians documented the social and health care burden. The heightened awareness led to a window of opportunity for policymaking. The strong network allowed a dominant frame of the problem to emerge and action to be undertaken. The technical nature of decision making and networks between research communities in health and agriculture allowed policy action to continue free from political interference, ‘under the radar’. Although strong political engagement is crucial, it might play such a key role only at specific stages of policymaking. At other times, institutions, key people and local leaders are needed to continue to drive effective action forward.

## Supplementary Material

Translated Abstracts
