# Supplementary material for: Policymaking ‘under the radar’: a case study of pesticide regulation to prevent intentional poisoning in Sri Lanka
Source: Health Policy Plan. 2013 Dec 20;30(1):56–67. doi: 10.1093/heapol/czt096 (PMC4287191; doi:10.1093/heapol/czt096)
Supplement: Translated Abstracts [file supp_30_1_56__index.html]

Policymaking ‘under the radar’: a case study of pesticide regulation to prevent intentional poisoning in Sri Lanka — Policymaking ‘under the radar’: a case study of pesticide regulation to prevent intentional poisoning in Sri Lanka — Translated Abstracts 

# Policymaking ‘under the radar’: a case study of pesticide regulation to prevent intentional poisoning in Sri Lanka

## Translated Abstracts

files

**Files in this Data Supplement:**

- Chinese Abstract - pdf file
- French Abstract - pdf file
- Spanish Abstract - pdf file
