# Supplementary material for: Policymaking ‘under the radar’: a case study of pesticide regulation to prevent intentional poisoning in Sri Lanka
Source: Health Policy Plan. 2013 Dec 20;30(1):56–67. doi: 10.1093/heapol/czt096 (PMC4287191; doi:10.1093/heapol/czt096)
Supplement: Translated Abstracts [file supp_czt096_ctz096_Chinese.pdf]

在“雷达监视”下的政策制定：通过对杀虫剂的监管从而防止斯里兰卡故意投毒事件的案例研究

研究背景

在斯里兰卡，自杀是重大公共安全问题之一，在 1995 年斯里兰卡是世界范围内自杀率最高的国家之一。从那时开始，整体自杀率的降低归因于一系列对杀虫剂监管的努力。我们调查了杀虫剂监管主要政策的产生，背景、重大事件以及政策的实行。

研究方法方

此研究作为更广泛的政策研究的一部分，政策研究包含两部分：历史性的记叙和解释性的 案例研究。本文通过深层访谈和文献综述来描述历史性部分。

研究结果

从访谈和文件数据中，我们梳理出关于政策行动和影响的时间轴和大事件。我们与 14 个关键的人员进行了访谈，并且划分了 4 个独特的政策阶段。在杀虫剂监管措施的早期阶段中，政治和经济方面的考虑占据主导的位置，监管措施在很大程度上受到外部因素的影响。第二阶段以当地政策的确立，当地利益相关者的参与，以及健康和农业之间不断延伸的关系作为标志。在第三阶段中，控制投毒自杀占据政策制定的主要位置；利益相关者，事实和政策制定三者之间存在更加密切的关系。第四阶段，也就是最近阶段，以当地大量的政策制定，人们对现实情况的了解，以及包括全球范围调查者在内的利益相关者强大的人际网络的合作作为标志。

研究结论

对使用杀虫剂故意投毒造成的极高自杀率的政策反应向我们展示了一个既特殊又成功的通过政策制定防止自杀的先例。它同时强调了政策措施发生在“雷达监视”的情况下，从而避免在中低收入国家改革中常出现的政策惰性
