# Supplementary material for: Policymaking ‘under the radar’: a case study of pesticide regulation to prevent intentional poisoning in Sri Lanka
Source: Health Policy Plan. 2013 Dec 20;30(1):56–67. doi: 10.1093/heapol/czt096 (PMC4287191; doi:10.1093/heapol/czt096)
Supplement: Translated Abstracts [file supp_czt096_czt096_French.pdf]

L’élaboration des politiques « sous surveillance » : étude de cas sur la régulation des pesticides afin d’éviter l’empoisonnement intentionnel au Sri Lanka

Accepté le 2 novembre 2013

Le suicide au Sri Lanka est un problème majeur de santé publique. En 1995, le pays avait le taux de suicide le plus élevé au monde. Depuis lors, le taux global de suicide a baissé largement dû aux efforts faits pour réglementer un certain nombre de pesticides. L’évolution, le contexte, les évènements et l’implantation des décisions politiques clés sont examinés de très près.

Méthodes

Cette étude a été entreprise dans le cadre d’une analyse plus large des mesures prises se divisant en deux parties, un récit historique et une étude de cas explicative. Nous décrivons dans cet article le contexte historique qui a entrainé une série d’interviews approfondies et l’analyse de documents.

Résultats

Grâce aux interviews et aux documents, nous avons pu tracer un historique et une chronologie des décisions et de l’influence politique. Les interviews ont été menées auprès de quatorze acteurs clés et nous avons pu identifier quatre phases politiques distinctes. La première phase concernant la réglementation sur les pesticides a été dominée par des considérations politiques et économiques et a connu de fortes pressions extérieures. La deuxième phase a été marquée par un renforcement des institutions locales, par le soutien de partenaires locaux et par des contacts plus nombreux entre le monde de la santé et le monde de l’agriculture. Pendant la troisième phase, les problèmes d’auto intoxication ont dominé l’agenda politique et dans le même temps des liens plus forts se sont développés entre les partenaires ainsi que des cas concrets pour l’élaboration de mesures. La quatrième phase, la plus récente, s’est caractérisée par une forte capacité locale à élaborer des mesures, nourries par des expériences concrètes et développée en collaboration avec un puissant réseau de partenaires, y compris des chercheurs internationaux.

Conclusions :

La réponse politique à ces taux extrêmement élevés de suicide par empoisonnement avec des pesticides est un très bon exemple d’élaboration de mesures pour lutter contre le suicide. Ceci met aussi en évidence les décisions politiques qui ont lieu « sous surveillance », et donc pour éviter l’inertie politique qui est souvent associée aux réformes dans les pays à faible et moyen revenu.

Mots clés : suicide, pesticides, analyse de l’élaboration des mesures, mesures basées sur du concret, politique de santé, agriculture, prévention, pays en développement.
