# Supplementary material for: Policymaking ‘under the radar’: a case study of pesticide regulation to prevent intentional poisoning in Sri Lanka
Source: Health Policy Plan. 2013 Dec 20;30(1):56–67. doi: 10.1093/heapol/czt096 (PMC4287191; doi:10.1093/heapol/czt096)
Supplement: Translated Abstracts [file supp_czt096_czt096_Spanish.pdf]

La formulación de políticas "bajo el radar": un estudio de caso de la regulación de los pesticidas para prevenir el envenenamiento intencional en Sri Lanka

Melissa Pearson, Anthony B. Zwi, Nicholas A. Buckley, Gamini Manuweera, Ravindra Fernando, Andrew H. Dawson y Duncan McDuie-Ra

Aceptado 2 de noviembre de 2013

Antecedentes

El suicidio en Sri Lanka es un importante problema de salud pública y en 1995 el país tuvo una de las tasas más altas de suicidio en todo el mundo. Desde entonces, las reducciones en las tasas globales de suicidio se han atribuido en gran parte a los esfuerzos para regular una serie de pesticidas. La evolución, el contexto, los eventos y la aplicación de las decisiones políticas clave alrededor de la regulación han sido examinadas.

Métodos

Este estudio se llevó a cabo como parte de un análisis más amplio de la política en dos partes: una narrativa histórica y un estudio de caso explicativo. Este artículo describe el componente de la narrativa histórica que se basó en entrevistas en profundidad y revisión de documentos.

Resultados

Una línea de tiempo y la cronología de las acciones de política e influencia fueron derivados de datos de entrevistas y de documentos. Catorce informantes clave fueron entrevistados y se identificaron cuatro fases políticas distintas. La etapa inicial de la regulación de pesticidas fue dominada por consideraciones políticas y económicas y fuertemente influenciada por factores externos. La segunda fase estuvo marcada por un período de construcción de instituciones locales, la participación de actores locales y los vínculos expandidos entre salud y agricultura. Durante la tercera fase, el problema del auto envenenamiento dominó la agenda política y se desarrollaron vínculos más estrechos entre las partes interesadas, las pruebas y la formulación de políticas. La cuarta y más reciente fase se caracterizó por una fuerte capacidad local para la formulación de políticas, respaldada por datos probatorios, desarrollada en colaboración con una

potente red de partes interesadas, incluidos los investigadores internacionales.

Conclusiones

La respuesta política a las tasas extremadamente altas de suicidio por envenenamiento intencional con pesticidas muestra un ejemplo único y exitoso de la formulación de políticas para prevenir el suicidio. También pone de relieve la acción política que tiene lugar "bajo el radar", evitando así la inercia política a menudo asociada con las reformas en los países de ingresos bajos y medios.

Palabras claves

suicidio, pesticidas, análisis de políticas, política basada en la evidencia, política de salud, agricultura, prevención, países en desarrollo

MENSAJES CLAVES

- La regulación de los pesticidas en Sri Lanka durante un período de 20 años redujo la mortalidad por suicidio y ofrece un ejemplo de la colaboración intersectorial para prevenir las muertes evitables.
- Se estableció una fuerte apropiación local del problema ya que los investigadores y los clínicos locales documentaron la carga social y al cuidado de la salud y esto condujo a una ventana de oportunidades para la formulación de políticas.
- Una fuerte red permitió que surgiera un marco dominante del problema y esto facilitó la acción a tomar.
- La naturaleza técnica de la toma de decisiones y las redes entre las comunidades de investigación en salud y agricultura permitió la acción política para continuar libre de injerencias políticas, "bajo el radar".

DOCUMENTO	FECHA DE RECIBO	OFICINA RESPONSABLE	DESPACHADO A:	
czt092	13/11/2013	Londres		
czt093	15/11/2013	Washington		
czt094	18/11/2013	Bogotá		
czt095	19/11/2013	Bogotá	London/Wash	22/11/2013
czt096	19/11/2013	Bogotá	London/Wash	23/11/2013
czt097	21/11/2013	Washington		
